# Efficient representation of boolean decision structures through Boolean function optimization

**DOI:** 10.3389/frai.2026.1801613

**Published:** 2026-06-19

**Authors:** Maddimsetti Srinivas, Debdoot Sheet

**Affiliations:** Department of Electrical Engineering, Indian Institute of Technology Kharagpur, Kharagpur, West Bengal, India

**Keywords:** Boolean decision structure, clustering, ESPRESSO algorithm, feature histogram, random forest

## Abstract

A binary decision tree (BDT) is stochastic and depth-dependent when inference is performed. The lower and upper bounds are derived from the minimum and maximum heights of the leaf nodes. The inherent randomness complicates BDT and random forest (RF) inference processes for fixed-rate streaming data. BDT is reformulated as a Boolean decision structure (BDS) in the proposed method to enable constant-time complexity. Optimized BDS (OBDS) is constructed by aggregating decision nodes exhibiting approximate boundary similarity. Further optimization of the Boolean function is achieved by applying the ESPRESSO algorithm on OBDS (EOBDS). Based on empirical evidence, BDS, OBDS, and EOBDS are statistically equivalent on BDT and BDT-based RF, and exhibit a constant time complexity of inference, regardless of BDT depth or number of BDTs.

## 1 Introduction

**Figure 1:**
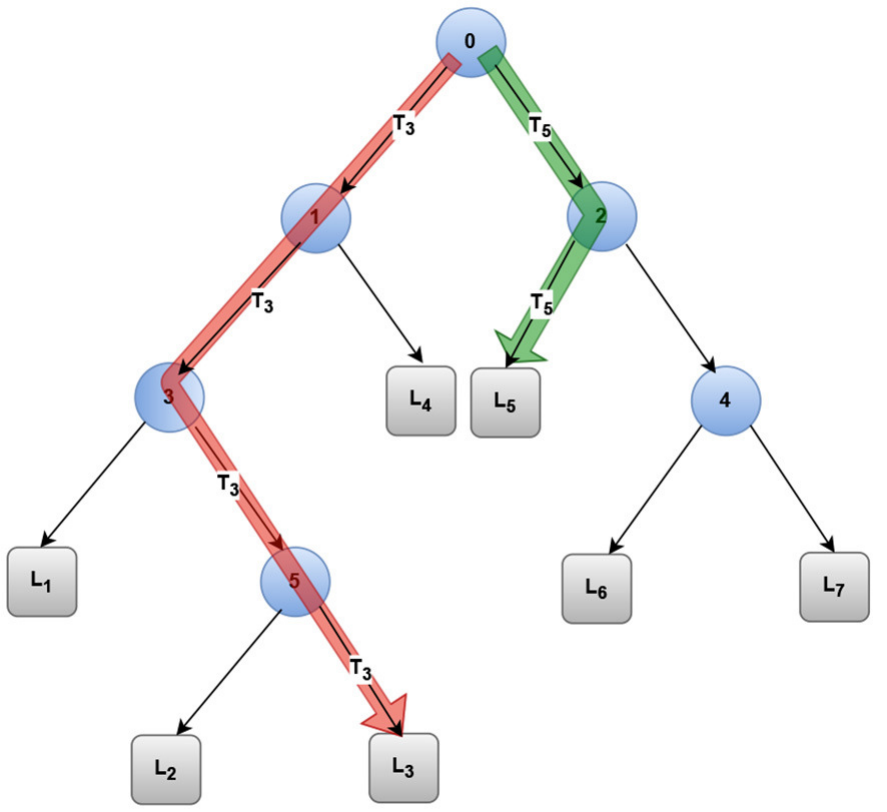
Visualization of a BDT. Circles represent internal decision nodes, while squares represent terminal leaf nodes. The shortest test paths are 0 → 1 → *L*_4_ and 0 → 2 → *L*_5_, while the longest are 0 → 1 → 3 → 5 → *L*_2_ and 0 → 1 → 3 → 5 → *L*_3_.

Binary decision tree (BDT) (Breiman et al., 1984; Quinlan, 1986) and random forest (RF) (Breiman, 2001; Ho, 1998; Criminisi et al., 2012) are widely used machine learning models due to their ease of implementation and interpretability. A BDT is typically visualized as a tree structure, where the root node serves as the starting point, internal nodes (circles) correspond to decision points, and terminal nodes (squares) represent final outcomes. Figure 1 shows a BDT with six decision nodes indexed by *n*∈{0, 1, …, 5}. At each decision node, a feature index *f*_*n*_ and a threshold θ_*n*_ define the axis aligned split function is written as in Equation 1:

$$S(v_{l};f_{n},\theta_{n})=\{\begin{array}{ll} 1 & \text{if }v_{f_{n}}\geq \theta_{n},\\ 0 & \text{otherwise}. \end{array}$$ (1)

The *l*^*th*^ sample in a *D*-dimensional space is denoted as $v_{l}=\left[v_{0},v_{1},\ldots ,v_{D-1}\right]\in {\mathbb{R}}^{D}$, where *v*_*d*_ denotes the *d*^*th*^ component of **v**_*l*_. Here, *f*_*n*_ is an index from the set {0, 1, …, *D*−1}, and *n*∈{0, 1, …, *N*−1}, where *N* is the total number of decision nodes in the BDT. During the evaluation of the split function *S*(·), if the condition is satisfied, the sample is directed to the right branch; otherwise, it proceeds to the left branch.

In a BDT, the parameter set is denoted by $D=\left\{\left(f_{0},\theta_{0}\right),\ldots ,\left(f_{N-1},\theta_{N-1}\right)\right\}$, where each pair represents the feature index and threshold associated with a decision node. The leaf

nodes of the BDT are described by $L=\left\{L_{0},\ldots ,L_{m},\ldots ,L_{M-1}\right\}$, where *L*_*m*_ corresponds to the *m*^*th*^ leaf node out of *M* total leaf nodes. Each leaf node selects a label from the set $W=\left\{\omega_{0},\ldots ,\omega_{c},\ldots ,\omega_{C-1}\right\}$, so that $L_{m}\in W$. Thus, a binary decision tree can be mathematically represented as a function $\hat{\omega }=g\left(v_{l}\right)$, where *g*:ℝ^*D*^↦{0, 1}^*C*^.

Each leaf node in a BDT is accessed by navigating from the root node along a specific path. In Figure 1, short and long traversal paths within a single BDT are illustrated. For instance, the shortest paths include moving from (*f*_0_, θ_0_) to (*f*_1_, θ_1_) and arriving at the leaf node *L*_4_, or transitioning from (*f*_0_, θ_0_) through (*f*_2_, θ_2_) to reach the leaf node *L*_5_. These paths can be mathematically described as: *T*_4_ = {(*f*_0_, θ_0_), (*f*_1_, θ_1_)} *T*_5_ = {(*f*_0_, θ_0_), (*f*_2_, θ_2_)}. Conversely, the longest traversal routes lead to the leaf nodes *L*_2_ and *L*_3_, following an identical sequence: *T*_2_ = *T*_3_ = {(*f*_0_, θ_0_), (*f*_1_, θ_1_), (*f*_3_, θ_3_), (*f*_5_, θ_5_)}. Let τ denote the average inference time per decision node, and *Q* the total traversal time. The minimum and maximum inference times are thus expressed as: *Q*_min_ = 2τ, *Q*_max_ = 4τ. Each node is associated with a specific test path *T*_*m*_, and every leaf node among the *M* nodes corresponds to a unique test path.

A machine learning model that processes streaming data (Huyen, 2022; Nakandala et al., 2020) should have a processing latency lower than that between successive samples in order to avoid delay due to resampling. The reduction of model complexity through various performance-preserving techniques (Li et al., 2020) is necessary to satisfy such requirements and reduce inference time (Prasad et al., 2024). As a result of this work, we propose that constant-time complexity inference can be achieved in BDT and RF using the following steps. The first two steps, Boolean decision structure (BDS) and optimized Boolean decision structure (OBDS), are published in Srinivas and Sheet (2024), and the minimization of OBDS is added as a third step.

1. The set of decision nodes in D is represented as a BDT. This representation is defined as Γ_BDS_ = {*x*_0_, …, *x*_*N*−1_}, where each pair (*f*_*n*_, θ_*n*_) maps to a Boolean variable *x*_*n*_. These Boolean variables correspond to the outcomes along specific test paths *T*_*m*_, expressed as products of Boolean terms. The overall Boolean function *F*_ω_ is then constructed using a Sum of Products (SOP) formulation, aggregating all paths that contribute to determining the truth value of a given class. This structure enables the inference process to operate efficiently, achieving predictable and constant time complexity.
2. To minimize the total number of Boolean variables within Γ_BDS_, similar groups of pairs (*f*_*n*_, θ_*n*_) in each BDT can be clustered together. Additionally, the set D representing the set of decision nodes of each BDT within an RF can be reorganized. This grouping and restructuring process leads to an OBDS, effectively reducing the overall number of Boolean variables required for representation.
3. Minimize the algebraic form of SOP using the ESPRESSO algorithm to reduce the constant time computational complexity further, thereby deriving the ESPRESSO algorithm on OBDS (EOBDS).

This paper is organized as follows. Section 2 presents the literature survey. Section 3 presents the method of constructing BDS, OBDS, and its refinement to form EOBDS. Section 4 shows the experiments and results and discussion is presented in Section 5, and we give complexity analysis in Section 6 and conclusion in Section 7.

## 2 Literature survey

Since the inception of artificial intelligence (AI), logic has played a fundamental role (McCarthy et al., 1960). A logical sentence semantics that allows probabilistic values on sentences has been presented as a straightforward generalization of the true or false semantics (Nilsson, 1986). Tractable Boolean circuits are enabling three modern applications of logic in AI: as a computational basis, for learning with combined data and symbolic knowledge, and for reasoning about machine learning systems (Darwiche, 2020). On the computation side, the machine models, like Bayesian networks, are transformed into Boolean formulas initially. Boolean formulas are compiled into tractable negation normal form (NNF) circuits (Darwiche and Marquis, 2002) by systems called knowledge compilers. Logic helps by excluding situations that contradict existing symbolic knowledge. This reduces the data required and makes the learned representations more robust (Kisa et al., 2014; Nishino et al., 2017). The third role of logic is to reason about the behavior of machine learning systems (Darwiche and Hirth, 2020).

The growing complexity of machine learning and probabilistic models, particularly deep neural networks and graphical models, has led to significant gains in accuracy in various domains, including computer vision, natural language processing, and decision-making systems. However, this complexity often comes at the cost of interpretability and tractability, which are essential for debugging, trust, accountability, and real-time inference. A central motivation for compiling such complex models into tractable representations, such as binary decision diagram (BDD) (Akers, 1978) and arithmetic circuit (AC) (Darwiche, 2021), is to enable efficient inference and logical reasoning. These representations are compact, structured, and can often support exact computations that are otherwise intractable on the original models.

The rapid growth of the internet of things (IoT) and the demand for on-device intelligence have driven the emergence of tiny machine learning (TinyML), which enables efficient inference on resource-constrained embedded systems. Foundational work in edge and mobile edge computing (Shi et al., 2016; Abbas et al., 2017) has established the vision and challenges of bringing computation closer to the data source, forming the basis for distributed intelligence. With the advent of specialized hardware such as the GreenWaves Application Processor 8 (GAP-8) system on chip (SoC) (Flamand et al., 2018), designed for AI at the edge, and hardware-aware model compression techniques such as hardware-aware automated quantization (HAQ) (Wang et al., 2019), TinyML has evolved into a practical paradigm for low-power deep learning. Frameworks like TensorFlow Lite Micro (David et al., 2021) have further accelerated this development by enabling deployment of machine learning models on microcontrollers with kilobyte-level memory footprints. Recent surveys (Dutta and Bharali, 2021; Doyu et al., 2021) highlight the growing TinyML ecosystem and its challenges in scalability, energy efficiency, and interoperability across heterogeneous devices. As a result, TinyML represents a significant step toward ubiquitous embedded intelligence, where machine learning inference occurs seamlessly at the extreme edge of the network, enabling real-time, privacy-preserving, and energy-efficient applications.

Recent research has focused extensively on optimizing the runtime performance of tree-based machine learning models through various hardware-aware and algorithmic approaches. Asadi et al. (2014) proposed runtime optimizations for tree-based models to reduce computational overhead during inference. Jo et al. (2013) investigated automatic vectorization techniques to accelerate tree traversal operations, and more recently, Prasad et al. (2022) introduced *Treebeard*, a compiler framework for optimizing decision tree inference. Xie et al. (2021) developed a tree structure-aware high-performance inference engine(Tahoe), optimized for graphics processing unit (GPU) execution, and Van Lunteren Van Lunteren, (2023) proposed adaptive parallelization strategies for decision-tree-based inference. These efforts collectively highlight the growing emphasis on leveraging hardware capabilities and compiler-level transformations to achieve high-performance decision tree inference. Despite these significant advances, most approaches still exhibit data-dependent execution paths that lead to variable inference latency. Therefore, achieving constant-time inference remains a critical research goal to ensure deterministic performance, particularly for real-time and embedded applications where latency predictability is essential.

## 3 Proposed method

### 3.1 Boolean decision structure (BDS)

Our approach converts a continuous feature *f*_*n*_ into a Boolean variable *x*_*n*_ using a threshold value θ_*n*_∈ℝ at a decision node within a BDT. Consider A as the dataset containing all samples, where |A| represents the total number of samples. From this dataset, a subset B is drawn with replacement, ensuring that B⊂A, and |B| indicates the sample count in this subset. Let |M| denote the minimum number of samples required at a decision node to apply a splitting function *S*(*v*_*i*_; *f*_*n*_, θ_*n*_). This relationship can be expressed mathematically as in Equation 2:

$$\begin{array}{l} \left|M|=\lambda |B\right| \end{array}$$ (2)

where λ∈(0, 1) serves as a complexity parameter, effectively regulating the size and depth of the BDT.

Consider $M_{X}$ as the set of data samples located at the parent node, while $M_{Y}$ and $M_{Z}$ represent the data samples directed to the left and right child nodes, respectively. The sizes of these sets satisfy the relationship $\left|M_{Y}|+|M_{Z}|=|M_{X}\right|$. The term *IG*(·) represents the information gain (IG), while $H\left(M_{X}\right)$ quantifies the entropy at the parent node across all classes. Similarly, $H\left(M_{Y}\right)$ and $H\left(M_{Z}\right)$ represent the entropy of the left and right child nodes, respectively. The entropy at the parent node can be mathematically expressed as in Equation 3:

$$\begin{array}{l} H\left(M_{X}\right)=-\overset{C-1}{\underset{c=0}{\sum }}\left\{p\left(\omega_{c}|M_{X}\right)\log_{2}\left(p\left(\omega_{c}|M_{X}\right)\right)\right\}\; \end{array}$$ (3)

Here, $p\left(\omega_{c}|M_{X}\right)$ indicates the probability of class ω_*c*_ within the set of class labels {ω_0_, …, ω_*C*−1_}, determined using the data samples from $M_{X}$. The information gain corresponding to a split threshold θ_*nd*_ is given by Equation 4:

$$\begin{array}{l} IG(\theta_{nd})=H({ℳ}_{X})-\frac{\left|{ℳ}_{Y}\right|}{\left|{ℳ}_{Y}|+|{ℳ}_{Z}\right|}H({ℳ}_{Y})\\ \;-\frac{\left|{ℳ}_{Z}\right|}{\left|{ℳ}_{Y}|+|{ℳ}_{Z}\right|}H({ℳ}_{Z}) \end{array}$$ (4)

This equation captures the reduction in entropy achieved through the split, thereby quantifying the effectiveness of the chosen threshold θ_*nd*_ in improving node purity.

**Figure 2:**
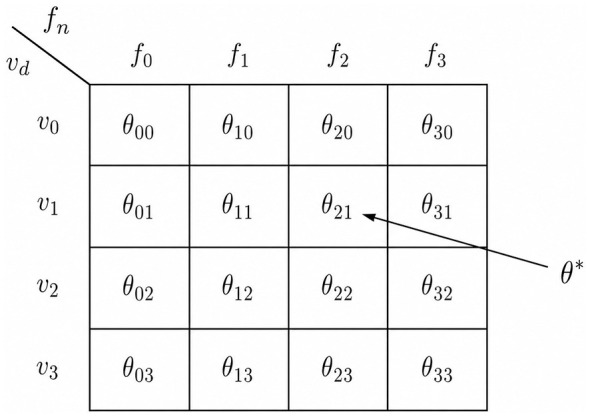
Optimal threshold selection process. The threshold value θ that results in the maximum IG is chosen as the optimal split point. In this scenario, if θ_21_ produces the highest IG among all possible threshold values, it is designated as the optimal split value θ^*^.

The primary splitting criterion for selecting both a feature index *f*_*n*_ and a threshold value θ_*n*_ at the *n*^*th*^ node, represented as (*f*_*n*_, θ_*n*_) using IG. For instance, suppose there are four features *f*_0_, *f*_1_, *f*_2_, *f*_3_ and four random threshold values *v*_0_, *v*_1_, *v*_2_, *v*_3_ associated with each feature, as depicted in Figure 2. These random threshold values are denoted as a set {θ_00_, …, θ_*nd*_, …, θ_*N*−1*D*−1_}, where θ_*nd*_ indicates the *d*^*th*^ random threshold value for the *n*^*th*^ feature. At each decision node, the algorithm identifies the threshold value θ that yields the highest information gain. The optimal threshold θ^*^ is selected from the set {θ_00_, …, θ_*N*−1*D*−1_} to ensure that the resulting information gain is maximized at the decision node.

In a BDT, the empirical distribution *p*(ω_*c*_, *L*_*m*_) represents the probability distribution over *C* possible classes at a specific leaf node *L*_*m*_. For a given sample **v**_*l*_, the posterior probability of belonging to class ω_*c*_ is denoted by $p\left(\hat{\omega }|v_{l},L_{m}\right)$. At the leaf node *L*_*m*_, the BDT employs the Maximum *a posteriori* (MAP) criterion to determine the most likely class label. Mathematically, this decision rule is expressed as in Equation 5:

$$\begin{array}{l} \hat{\omega }\left(v_{l}\right)=\underset{c}{\arg\max}\left\{p\left(\omega_{0}|v_{l}\right),\ldots ,p\left(\omega_{c}|v_{l}\right),\ldots ,p\left(\omega_{C-1}|v_{l}\right)\right\} \end{array}$$ (5)

Here, $\hat{\omega }\left(v_{l}\right)$ signifies the predicted class label for the sample **v**_*l*_, selected based on the class with the highest posterior probability.

**Figure 3:**
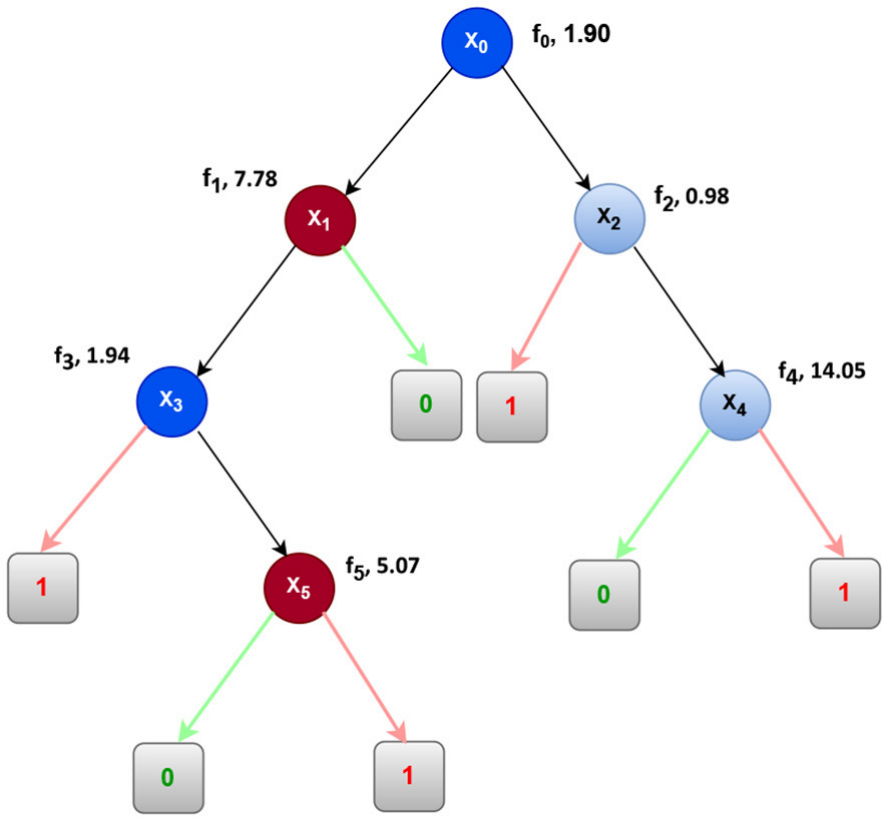
A BDT can be transformed into a Boolean function representation. Decision nodes are identified by features *f*_*n*_ and split values θ_*n*_, which are accompanied by Boolean variables *x*_*n*_. In other words, *x*_*n*_ = *TRUE* if *f*_*n*_≥θ_*n*_, and *x*_*n*_ = *FALSE* if otherwise. One feature can lead to multiple Boolean variables. As an example, feature *f*_1_ is the source of both features *x*_0_ and *x*_3_; and feature *f*_1_ is the source of both features *x*_1_ and *x*_5_. As a result of this process, a total number of Boolean variables is generated, corresponding to the number of internal decision nodes in the BDT. The blue circle represent the nodes belong to the same (*x*_0_ and *x*_3_), similarly for the brown circle (*x*_1_ and *x*_5_).

Consider a decision tree where each internal node splits a feature *f*_*n*_ into two branches based on a threshold value θ_*n*_. Each branch then generates a Boolean variable *x*_*n*_ or its complement ${\bar{x}}_{n}$. A single feature can yield more than one Boolean variable on the same test path. For instance, *x*_0_ and *x*_3_ are both derived from *f*_0_ but with different thresholds θ_0_ and $\theta_{0}^{\prime }$. Each leaf node *L*_*m*_ corresponds to a unique path from the root to the leaf, representing a specific combination of Boolean variables. This path, or test path, can be expressed as a logical term *T*_*m*_. A two-class problem can be demonstrated by Figure 3, in which Boolean function ω_0_ realized as in Equation 6

$$\begin{array}{l} F_{\omega_{0}}=(\neg x_{0}\wedge \neg x_{1}\wedge x_{3}\wedge \neg x_{5})\vee (\neg x_{0}\wedge x_{1})\\ \;\vee (x_{0}\wedge x_{2}\wedge \neg x_{4}) \end{array}$$ (6)

Similarly as in Equation 7

$$\begin{array}{l} F_{\omega_{1}}=(\neg x_{0}\wedge \neg x_{1}\wedge \neg x_{3})\vee (\neg x_{0}\wedge \neg x_{1}\wedge x_{3}\wedge x_{5})\\ \;\vee (x_{0}\wedge \neg x_{2})\vee (x_{0}\wedge x_{2}\wedge x_{4}) \end{array}$$ (7)

The inference of Boolean functions is constant time-complex, unlike that of traditional BDTs.

### 3.2 Optimized Boolean decision structure (OBDS)

For each feature in the dataset, we construct a histogram using threshold values $D=\left\{\left(f_{0},\theta_{0}\right),\ldots ,\left(f_{N-1},\theta_{N-1}\right)\right\}$ employed at the decision nodes of all BDTs within the RF. The resulting histogram for the *n*^th^ feature, denoted as *h*(*f*_*n*_), can be effectively modeled using a Gaussian mixture model (GMM). This statistical approach, parameterized by **β**, allows us to capture the underlying distribution of feature thresholds and identify significant patterns within the data. Let α_0_, …, α_*Z*−1_ represent the mixing probabilities, μ_0_, …, μ_*Z*−1_ the corresponding means, and **β***_0_*, …, **β**_*Z*−1_ the corresponding parameters of the components Π_0_, …, Π_*Z*−1_. These parameters can be estimated using unsupervised learning techniques for finite mixture models, as proposed by Figueiredo and Jain (2002). For each decision node feature pair (*f*_*n*_, θ_*n*_) in D, a feature histogram *h*(*f*_*n*_) is created using θ_*n*_. For each feature histogram *h*(*f*_*n*_), the probability is computed as a weighted sum of conditional probabilities as written in Equation 8,

$$\begin{array}{l} p\left(h\left(f_{n}\right)|\beta \right)=\overset{Z-1}{\underset{i=0}{\sum }}\alpha_{i}\cdot p\left(h\left(f_{n}\right)|\beta_{i}\right) \end{array}$$ (8)

where the extracted components are Π_0_, …, Π_*Z*−1_ with corresponding means μ_0_, …, μ_*Z*−1_. Then, for each test path *T*_*m*_ in T, the set of test paths in a BDS, and each component Π_*z*_ in H, the set of components, all pairs (*f*_*n*_, θ_*n*_) and $\left(f_{n}^{\prime },\theta_{n}^{\prime }\right)$ in *T*_*m*_ are compared. If θ_*n*_ and $\theta_{n}^{\prime }$ belong to Π_*z*_, they are replaced with the mean value μ_*z*_. This iterative process ensures optimization across the decision structure. Thus, Boolean function for ω_0_ OBDS represented as in Equation 9

$$\begin{array}{l} F_{\omega_{0}}^{*}=(\neg x_{0}\wedge \neg x_{1}\wedge \neg x_{5})\vee (\neg x_{0}\wedge x_{1})\\ \;\vee (x_{0}\wedge x_{2}\wedge \neg x_{4}) \end{array}$$ (9)

similarly as in Equation 10, for ω_1_

$$\begin{array}{l} F_{\omega_{1}}^{*}=(\neg x_{0}\wedge \neg x_{1})\vee (\neg x_{0}\wedge \neg x_{1}\wedge x_{5})\\ \text{​​ }\vee (x_{0}\wedge \neg x_{2})\vee (x_{0}\wedge x_{2}\wedge x_{4}) \end{array}$$ (10)

BDS is modified to remove the Boolean variable *x*_3_ after eliminating redundant threshold values.

### 3.3 ESPRESSO algorithm on OBDS (EOBDS)

We apply the ESPRESSO algorithm (Brayton et al., 1984) to the Boolean functions $F_{\omega_{0}}^{*}$ and $F_{\omega_{1}}^{*}$ of OBDS. The goal of applying the ESPRESSO algorithm on OBDS is to minimize the Boolean functions further and obtain EOBDS. Therefore, we obtain modified versions of $F_{\omega_{0}}^{**}$ and $F_{\omega_{1}}^{**}$ are in Equations 11 and 12 as follows:

$$\begin{array}{l} F_{\omega_{0}}^{**}=\left(\neg x_{0}\wedge \neg x_{5}\right)\vee \left(\neg x_{0}\wedge x_{1}\right)\vee \left(x_{0}\wedge x_{2}\wedge \neg x_{4}\right) \end{array}$$ (11)

$$\begin{array}{l} F_{\omega_{1}}^{**}=\left(\neg x_{0}\wedge \neg x_{1}\right)\vee \left(x_{0}\wedge \neg x_{2}\right)\vee \left(x_{0}\wedge x_{4}\right) \end{array}$$ (12)

These modified Boolean functions represent the EOBDS for the given BDT. ESPRESSO reduces the complexity of OBDS and can lead to improved computational efficiency with the same accuracy as BDS.

## 4 Experimental settings

We used Python-based software tools for the implementation. A key component was the gmm-mml library ^1^, which we employed to model the feature histograms using unsupervised learning of GMM. Our experimentation encompassed two distinct datasets: image datasets and publicly available datasets from the University of California, Irvine Machine Learning (UCI ML) Repository^2^. We used a machine with an Intel(R) Core(TM) i5-4570 CPU at 3.20GHz, running Ubuntu 20.04 LTS, and featuring 32 GB of RAM for all experiments.

### 4.1 Training and testing sets

Our models were rigorously tested using a fivefold stratified cross-validation scheme. For each dataset, 80% of the samples were allocated to training sets, while the remaining 20% were reserved for testing. To introduce variability and enhance model robustness, we employed the bagging technique, randomly sampling 90% of the training data with replacement for each tree in the ensemble. The RF is constructed with 100 BDTs at a specified λ varies from 0.3 to 0.7 depends on the dataset. For each fold, we constructed the ensemble of BDSs as a Boolean forest (BF), the ensemble of OBDSs as an optimized Boolean forest (OBF), and the ensemble of EOBDSs as ESPRESSO on OBF (EOBF) with designated training set. Subsequently, these models were evaluated on their corresponding test set. A comprehensive assessment of the performance of all models was computed using average accuracy and standard deviation over the five folds.

## 5 Results and discussion

Moreover, we calculated the accuracy of the proposed method on UCI ML datasets as shown in Table 1. Furthermore, reduction in Boolean variables, two input AND and OR operations were analyzed for UCI ML datasets to calculate model complexity.

**Table 1:** Number of samples (|A|) and features (*D*) for each dataset.

Dataset	*D*	|A|	*C*	Samples/class	Features
wdbc	30	568	2	[211, 357]	Real
Bank	4	1,371	2	[761, 610]	Real
Haberman	3	305	2	[224, 81]	Integer
Iono	33	350	2	[224, 126]	Integer, real
Parkinsons	22	194	2	[48, 146]	Real
Pima	8	767	2	[500, 267]	Integer, real
Sonar	60	208	2	[111, 97]	Real
Mammograph	5	829	2	[427, 402]	Integer
Transfusion	4	747	2	[570, 177]	Real
Spambase	57	4,601	2	[2,788, 1,813]	Integer, real
Iris	4	149	3	[49, 50, 50]	Real
Wine	13	177	3	[58, 70, 49]	Integer, real
Balance	4	625	3	[288, 49, 288]	Categorical
Glass	9	214	6	[70, 76, 17, 13, 9, 29]	Real
Breast tissue	9	106	6	[21, 15, 18, 16, 14, 22]	Real
Zoo	16	101	7	[41, 20, 5, 13, 4, 8, 10]	Categorical, integer

Datasets such as wdbc, bank, haberman, and spambase are binary, while iris, wine, and zoo contain more than two classes. Most datasets have real-valued features, with some containing categorical or mixed types.

### 5.1 UCI ML datasets

A comparison of RF, RF-V, BF, OBF, and EOBF shown in Table 2 using UCI ML datasets. The Table highlights a wide range of accuracy across the UCI ML datasets of different sample sizes and dimensionality. In many instances, the differences in accuracy between naive RF and Boolean structures are relatively small, indicating that model size can be effectively reduced without significantly impacting performance. We performed a Friedman test across 16 datasets to compare the five models. The test indicated statistically significant differences among the models (*p* < 0.05) as shown in Table 2.

**Table 2:** Accuracy (%) with Standard Deviation (Mean ± Std) across datasets averaged over five folds.

Dataset	RF	RF-V	BDS	OBDS	EOBDS
wdbc	92.31 ± 2.06	92.09 ± 1.90	92.31 ± 1.94	92.75 ± 1.81	92.75 ± 1.81
Bank	90.37 ± 1.72	88.33 ± 2.63	88.04 ± 1.99	87.16 ± 2.95	87.96 ± 3.58
Haberman	73.44 ± 0.73	73.44 ± 0.73	73.44 ± 0.73	73.44 ± 0.73	73.44 ± 0.73
Iono	90.86 ± 2.17	90.00 ± 3.50	89.14 ± 3.86	89.14 ± 3.99	85.43 ± 4.78
Parkinsons	82.97 ± 3.09	82.46 ± 2.96	80.93 ± 1.33	80.92 ± 2.39	80.92 ± 2.39
Pima	65.19 ± 0.23	65.19 ± 0.23	65.19 ± 0.23	65.19 ± 0.23	65.19 ± 0.23
Sonar	76.39 ± 7.63	75.44 ± 7.56	75.91 ± 6.71	76.88 ± 6.43	76.40 ± 7.20
Mammograph	81.91 ± 2.70	81.31 ± 3.32	81.43 ± 3.08	81.07 ± 2.40	81.07 ± 2.71
Transfusion	76.31 ± 0.28	76.31 ± 0.28	76.31 ± 0.28	76.31 ± 0.28	76.44 ± 0.25
Spambase	90.15 ± 0.68	89.57 ± 1.13	89.17 ± 1.29	89.07 ± 1.21	89.46 ± 1.15
Iris	91.93 ± 5.61	87.89 ± 5.19	89.26 ± 3.64	87.91 ± 3.85	87.91 ± 3.85
Wine	93.21 ± 5.19	89.24 ± 6.54	88.67 ± 7.32	89.25 ± 7.13	88.68 ± 7.85
Balance	85.30 ± 1.82	84.49 ± 2.80	85.03 ± 5.21	84.76 ± 4.00	84.50 ± 6.04
Glass	59.41 ± 4.00	57.64 ± 5.86	55.29 ± 8.03	55.88 ± 7.06	54.69 ± 7.18
Breast tissue	58.73 ± 2.75	55.56 ± 5.50	53.97 ± 2.75	53.97 ± 2.75	53.97 ± 2.75
Zoo	84.00 ± 4.18	77.00 ± 5.70	75.00 ± 5.00	75.00 ± 5.00	76.00 ± 4.18

A Friedman test conducted over 16 datasets indicates statistically significant differences among the models (χ^2^ = 17.6216, *p* = 0.0015 < 0.05). Based on the *p* value, these five models are not statistically equivalent.

#### 5.1.1 Complexity analysis

**Figure 4:**
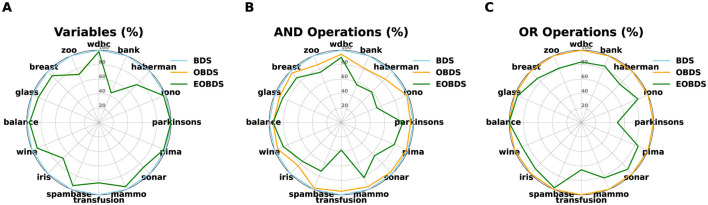
Dataset-wise complexity visualization. **(A)** Complexity levels for BF, **(B)** complexity levels for OBF, and **(C)** complexity levels for EOBF. Dataset labels are placed around the circumference of the chart, with polygon areas indicating the relative complexity.

Figure 4 illustrates the presence of multiple axes, each corresponding to a distinct dataset. A polygonal shape is formed by connecting the points on each axis to represent the proportion of variables or logical operations for every dataset. The space and operational complexity of the model across various datasets is depicted through each polygon. The blue line and shaded region represent the BF, positioned on the outermost circular boundary, calculated as $\frac{\left|\Gamma_{\text{bds}}\right|}{\left|\Gamma_{\text{bds}}\right|}\times 100\%$. Similarly, the orange line and its corresponding area represent the OBF, derived using $\frac{\left|\Gamma_{\text{obds}}\right|}{\left|\Gamma_{\text{bds}}\right|}\times 100\%$. The green line and shaded area correspond to the EOBF, expressed as $\frac{\left|\Gamma_{\text{eobds}}\right|}{\left|\Gamma_{\text{bds}}\right|}\times 100\%$. Each class is mapped along radial spokes originating from the center of the plot. The percentage representation of variables for every model is displayed as individual points on these spokes, with smooth connecting lines forming curves for each dataset. This visualization enables straightforward comparisons across the different models.

Figure 4A displays the percentage of Boolean variables in each model. Figures 4B, C show the percentages of two input AND gates and OR gates required for each model, respectively. The blue polygon represents BF, the orange polygon represents OBF, and the green polygon represents EOBF. Let us define ▽_*bds*_, ▽_*obds*_, and ▽_*eobds*_ as the number of AND operations corresponding to the BF, OBF, and EOBF models, respectively. For comparison, the relative percentages are calculated as $\frac{{▽}_{bds}}{{▽}_{bds}}\times 100\%$ for BF, $\frac{{▽}_{obds}}{{▽}_{bds}}\times 100\%$ for OBF, and $\frac{{▽}_{eobds}}{{▽}_{bds}}\times 100\%$ for EOBF. Similarly, let △_*bds*_, △_*obds*_, and △_*eobds*_ represent the number of OR operations in BF, OBF, and EOBF models, respectively. These metrics allow a standardized comparison of logical operations across the three models.

### 5.2 Inference times computation on UCIML datasets

The inference time of BF is the time taken to evaluate the Boolean functions of all the refers to the time required to evaluate the Boolean functions of all inference times for OBF and EOBF are computed. We observe a reduction in the inference time of OBF and EOBF from BF since we have obtained a reduction in the variables with OBF compared to BF, since we have achieved a decrease in the size of EOBF. Table 3 presents an inference time comparison across three approaches: BF, OBF, and EOBF, measured in milliseconds (*ms*) across multiple datasets. The results show a clear trend where EOBF consistently achieves the lowest inference time, followed by OBF and then BF.

**Table 3:** Comparison of inference times for BF, OBF, and EOBF averaged over five folds.

Dataset	BF (ms)	OBF (ms)	EOBF (ms)
wdbc	57.61 ± 3.09	54.11 ± 2.48	28.24 ± 1.64
Bank	47.57 ± 1.48	42.77 ± 2.56	20.56 ± 0.74
Haberman	16.99 ± 4.24	14.82 ± 3.36	5.13 ± 1.12
Iono	108.19 ± 3.31	100.83 ± 1.60	36.81 ± 1.61
Parkinsons	22.92 ± 1.22	21.50 ± 1.32	12.09 ± 0.55
Pima	32.98 ± 1.11	31.71 ± 1.27	12.57 ± 0.66
Sonar	28.35 ± 0.86	27.82 ± 1.71	12.01 ± 1.23
Mammograph	47.53 ± 3.23	43.38 ± 2.86	22.57 ± 1.48
Transfusion	38.88 ± 1.15	38.33 ± 2.27	9.94 ± 0.35
Spambase	1007.25 ± 12.44	997.66 ± 39.70	441.71 ± 11.73
Iris	9.05 ± 0.49	8.37 ± 0.57	4.54 ± 0.21
Wine	15.79 ± 0.72	14.88 ± 0.37	8.11 ± 0.19
Balance	38.46 ± 1.23	37.19 ± 1.16	16.24 ± 0.37
Glass	38.53 ± 1.86	36.12 ± 2.05	19.89 ± 0.49
Breast tissue	11.13 ± 0.98	10.72 ± 1.04	6.11 ± 0.58
Zoo	13.29 ± 0.53	12.80 ± 0.49	6.33 ± 0.20

For each dataset, 80% of the samples were allocated to training sets, while the remaining 20% were reserved for testing. Values are reported as mean ± standard deviation (ms).

### 5.3 Performance evaluation on image datasets

Details regarding the convolutional neural network (CNN) training process can be found in the Supplementary material of Srinivas and Sheet (2024). The RF contains 400 BDTs, and the complexity factor (λ) was set to 0.2. Table 4 presents a comparative analysis of accuracy (%) along with standard deviation for all models: RF, RF-V, BF, OBF, EOBF, and CNN. The results are reported for four widely used benchmark datasets: MNIST, FMNIST, KMNIST, and CIFAR-10, with features extracted from the *conv layer-10*. We performed a Friedman test to compare the performance of RF, RF-V, BF, OBF, EOBF, and CNN across four datasets. The results indicate statistically significant differences among the models (*p* < 0.05).

**Table 4:** Accuracy (%) comparison across datasets for RF, RF-V, BF, OBF, EOBF, and CNN.

Dataset	RF	RF-V	BF	OBF	EOBF	CNN
MNIST	94.29 ± 1.04	89.39 ± 3.41	92.79 ± 2.13	92.79 ± 2.13	92.79 ± 2.13	98.51
FMNIST	86.36 ± 2.41	81.10 ± 1.36	85.74 ± 2.34	86.11 ± 2.26	86.11 ± 2.26	91.22
KMNIST	86.88 ± 2.81	80.88 ± 4.64	84.13 ± 2.10	84.50 ± 1.47	84.25 ± 1.26	91.95
CIFAR-10	79.88 ± 0.01	74.00 ± 1.33	78.80 ± 1.31	79.20 ± 0.84	79.20 ± 0.84	85.36

Features extracted from *conv layer-10*. A Friedman test across the four datasets indicates statistically significant differences among the models (χ^2^ = 19.5522, *p* = 0.001516 < 0.05).

On the MNIST dataset, CNN achieves the highest accuracy at 98.51%, followed by RF (94.29 ± 1.04) and BF, OBF, and EOBF models, all performing equally at 92.79 ± 2.13. For the FMNIST dataset, CNN again outperforms other models with an accuracy of 91.22%, while OBF and EOBF achieve comparable performance at 86.11 ± 2.26, slightly outperforming BF (85.74 ± 2.34) and RF-V (81.10 ± 1.36). In the KMNIST dataset, CNN remains the top performer with an accuracy of 91.95%, while RF achieves 86.88 ± 2.81, and OBF and EOBF maintain close scores at 84.50 ± 1.47 and 84.25 ± 1.26, respectively. Finally, CNN achieves 85.36% on the CIFAR-10 dataset, outperforming RF (79.88 ± 0.01) and BDS (78.80 ± 1.31), and OBF and EOBF perform similarly at 79.20 ± 0.84. These results highlight that CNN consistently achieves the highest accuracy across all datasets, while OBF and EOBF provide competitive performance, demonstrating their effectiveness in optimizing Boolean Decision Structures for classification tasks. Unlike conventional knowledge distillation (KD) that distills output distributions, we transfer the internal learned structure from a deep model to a symbolic logic-based model.

## 6 Complexity analysis of BDT and BDS

### 6.1 Inference complexity of BDT

In a BDT, inference is performed by traversing a path from the root node to a leaf node. At each internal node, a decision function evaluates a feature-threshold pair (*f*_*n*_, θ_*n*_) and directs the sample to either the left or right subtree. Let *h* denote the height of the tree. Since one decision is evaluated per level until a leaf node *L*_*m*_ is reached, the inference cost is proportional to the path length as written in Equation 13:

$$\begin{array}{l} T_{\text{BDT}}=\Theta \left(h\right) \end{array}$$ (13)

The exact complexity depends on the tree structure. In the best case, inference follows the shortest valid root-to-leaf path, giving the lower bound as written in Equation 14:

$$\begin{array}{l} T_{\text{BDT}}=\Omega \left(\log N\right) \end{array}$$ (14)

for a balanced tree, where *N* is the number of internal nodes. In the worst case, inference follows the longest path in a highly skewed tree, giving the upper bound as written in Equation 15:

$$\begin{array}{l} T_{\text{BDT}}=O\left(N\right) \end{array}$$ (15)

Thus, the inference complexity of a BDT is bounded as written in Equation 16:

$$\begin{array}{l} \Omega \left(\log N\right)\leq T_{\text{BDT}}\leq O\left(N\right) \end{array}$$ (16)

It is important to note that BDT inference is inherently sequential, as each decision depends on the outcome of the previous node. Consequently, parallel evaluation across different paths is not feasible.

### 6.2 Inference complexity of BDS

The BDS transforms the BDT into a Boolean function representation. Each decision node (*f*_*n*_, θ_*n*_) is mapped to a Boolean variable as written in Equation 17:

$$\begin{array}{l} x_{n}=I\left(f_{n}\geq \theta_{n}\right) \end{array}$$ (17)

where *I*(·) denotes the indicator function, which returns 1 when the condition is true and 0 otherwise. Each root-to-leaf path is represented as a conjunction of Boolean literals as written in Equation 18:

$$\begin{array}{l} T_{m}=\underset{k\in I_{m}}{\wedge }x_{k}^{\alpha_{k}} \end{array}$$ (18)

where *I*_*m*_⊆{0, 1, …, *N*−1} denotes the index set of Boolean variables involved in the *m*^th^ path. In other words, *I*_*m*_ contains the indices of all decision nodes that appear along the root-to-leaf path *T*_*m*_. The exponent α_*k*_∈{0, 1} determines whether the literal is taken in its original or complemented form, such that $x_{k}^{1}=x_{k}$ and $x_{k}^{0}=\neg x_{k}$. Thus, each path *T*_*m*_ represents a conjunction of literals corresponding to the decisions encountered along that path. The overall classification function is expressed in SOP form as written in Equation 19:

$$\begin{array}{l} F_{\omega }=\underset{m\in M}{\vee }T_{m} \end{array}$$ (19)

The inference process in the BDS can be decomposed into three stages. First, all Boolean variables *x*_*n*_ corresponding to decision nodes are computed, which requires *O*(*N*) operations. Second, each decision path *T*_*m*_ is evaluated as a conjunction of Boolean literals, where each path contains at most *K* literals; this step incurs a computational cost of O(|T|·K). Here, T denotes the set of all decision paths that contribute to the classification, and T represents the total number of such paths. Finally, the outputs of all path terms are aggregated using a logical OR operation, requiring O(|T|) time. Combining these steps, the overall sequential inference complexity is given by $T_{\text{BDS}}=O\left(N+|T|\cdot K\right)$.

After training, the structure of the BDS becomes fixed, meaning that the number of Boolean variables *N*, the number of decision paths |T|, and the maximum path length *K* remain constant. These quantities are determined during the training phase and do not vary with the size or nature of the input data during inference. As a result, the computational steps required to evaluate the Boolean variables, compute the path terms, and aggregate the final decision are all bounded by fixed constants. Therefore, the inference complexity of the BDS does not scale with input size and can be expressed as constant time in Equation 20:

$$\begin{array}{l} T_{\text{BDS}}=O\left(1\right) \end{array}$$ (20)

## 7 Conclusion

The BDT has been converted into BDS so that constant-time inference can be performed. We created Boolean variables by converting the real values of the features in a BDT into binary values. Each feature in the dataset is then represented by a feature histogram using feature split values at the internal decision nodes in the RF model. The generated feature histogram for each feature is fit into a GMM to eliminate near-split values to reduce the space complexity. Additionally, the ESPRESSO algorithm is operated on the OBF for further optimization and streamlined logical operations to reduce the operational complexity. Streaming data applications can be made latency-free by using constant-time BDTs. Furthermore, we will work on simplifying deep learning models by utilizing optimized Boolean structures.

## Footnotes

1
https://github.com/abriosi/gmm-mml

2
https://archive.ics.uci.edu/ml/datasets
